# Analysis of blood metabolite characteristics at birth in preterm infants with bronchopulmonary dysplasia: an observational cohort study

**DOI:** 10.3389/fped.2024.1474381

**Published:** 2024-10-31

**Authors:** Yanping Guo, Jingjing Chen, Zhen Zhang, Chang Liu, Jiamin Li, Ying Liu

**Affiliations:** Department of Pediatrics, Peking University Shenzhen Hospital, Shenzhen, China

**Keywords:** bronchopulmonary dysplasia, preterm birth, blood, metabolomics, cohort study

## Abstract

**Background:**

To analyze the characteristics of blood metabolites within 24 h after birth in preterm infants with bronchopulmonary dysplasia (BPD) and to identify biomarkers for predicting the occurrence of BPD.

**Methods:**

Dried blood spots (DBS) were collected at birth from preterm infants with gestational age (GA) of less than 32 weeks in the cohort. The infants were divided into the BPD group and non-BPD group based on whether they eventually developed BPD. Dried blood spot filter papers were prepared from venous blood collected within the first 24 h of life. Metabolites were measured using liquid chromatography-tandem mass spectrometry (LC-MS/MS) and analyzed using the R software package.

**Results:**

DBS samples from 140 infants with the GA < 32 weeks were used in the study, with 4 infants who died being excluded. Among the remaining 136 preterm infants, 38 developed BPD and 98 did not. To control for GA differences, we conducted a subgroup analysis. In the GA 24^+4^–27^+6^ weeks subgroup, we observed a significant decrease in histidine levels and the ornithine/citrulline ratio in the BPD group. Additionally, the ratios of acylcarnitines C3/C0 and C5/C0 were also significantly reduced.

**Conclusions:**

Metabolic markers in DBS within 24 h after birth are promising for predicting the occurrence of BPD in preterm infants with GA < 28 weeks.

**Clinical Trial Registration:**

[https://www.chictr.org.cn/], identifier [ChiCTR2100048293, ChiCTR2400081615].

## 1 Introduction

Bronchopulmonary dysplasia (BPD) is a common chronic lung disease among premature infants. The survival rate of extremely preterm infants has significantly improved with advances in medical care, rising from 56.4% in 2010 to 67.1% in 2019 (1). However, the incidence of BPD has not noticeably decreased and remains high (2, 3). BPD not only prolongs hospital stays and increases medical costs for preterm infants but also results in adverse short- and long-term respiratory and neurological outcomes, and can even lead to death (4–8). Due to the diverse clinical symptoms of BPD and its susceptibility to various factors, there is currently a lack of objective criteria for accurately predicting its mortality and morbidity rates (9–12). Therefore, early prediction of BPD in high-risk preterm infants remains a key focus and challenge in current research.

Research also suggests that metabolic reprogramming emerges as a significant characteristic at the onset of BPD, primarily characterized by abnormalities in glucose, lipid, amino acid, and other metabolic pathways (13). Thus, utilizing metabolomic techniques to identify biomarkers in early-life biological samples from preterm infants (such as umbilical cord blood, urine, tracheal aspirates, or blood) may rapidly detect potential metabolic disturbances and identify unique metabolites for early prediction of BPD (14–16). Early identification of newborns at risk of developing BPD, coupled with timely targeted interventions, can assist in preventing and reducing the severity of the condition.

Metabolomics entails a comprehensive and systematic analysis of a variety of small molecule metabolites through high-throughput techniques. It reveals the distinctive metabolic characteristics of organisms, thus providing a method to discern physiological or pathological states (17). Currently, metabolomics is widely utilized in the field of neonatology, helping in the early identification and diagnosis of various diseases (18, 19). An increasing number of researchers are exploring the metabolomics of BPD. We conducted a systematic evaluation of differential metabolites between BPD and non-BPD preterm infants, highlighting the current absence of consistent metabolic markers for early BPD prediction. Therefore, we collected dried blood spots (DBS) from preterm infants with GA < 32 weeks within 24 h after birth. Using liquid chromatography-tandem mass spectrometry (LC-MS/MS), we performed targeted measurements of metabolites with the aim of identifying differential metabolites in preterm infants with BPD and uncovering circulating biomarkers for the early prediction of BPD.

## 2 Materials and methods

### 2.1 Study design and data collection

Data collection comes from a single-center prospective observational cohort study, “Cohort Study on the Impact of Vitamin D Deficiency on Bronchopulmonary Dysplasia in Preterm Infants,” registered at https://www.chictr.org.cn/(ChiCTR2100048293), and a multicenter prospective open observational cohort study, “Online Registration of Bronchopulmonary Dysplasia: A Multicenter, Prospective, Open, Observational Cohort Study,” registered at https://www.chictr.org.cn/(ChiCTR2400081615). From December 2019 to December 2023, preterm infants with GA < 32 weeks, born and admitted to the Neonatal Intensive Care Unit (NICU) at our hospital, with a NICU comprising 18 beds, were included in the study. The exclusion criteria were: (1) Congenital lung malformations and diaphragmatic hernia; (2) Severe congenital heart disease (excluding small ventricular septal defects, atrial septal defects, or patent ductus arteriosus); (3) Chromosomal abnormalities and genetic metabolic disorders; (4) Hospitalization for less than 28 days; and (5) Incomplete data. All patients had detailed records of their birth conditions, maternal pregnancy history, and treatments. Within 24 h of birth, prior to the initiation of parenteral nutrition, three drops of venous blood were collected to create dried blood spot filter papers. These papers were analyzed for targeted metabolite measurement using LC-MS/MS, including 13 amino acids, 31 acylcarnitines, and their ratios, totaling 86 analytes. BPD is diagnosed based on the 2018 criteria of the National Institute of Child Health and Human Development (NICHD) (20). Patients are divided into BPD and non-BPD groups to compare differences in metabolites between the two groups. This study was approved by the Ethics Committee of our hospital, and informed consent was obtained from the guardians of the subjects [PKU Shenzhen Hospital Ethics Approval (Research) (2019) No. 019 and (2024) No. 005].

### 2.2 Collection of dried blood spot specimens

During routine blood sampling shortly after birth, three drops of venous blood were collected and placed onto the blood circles of a collection card, ensuring that the patient's information matched that on the informed consent form. The blood drops were allowed to naturally permeate through to the back of the filter paper. Once one blood circle was fully saturated, the next drop was placed on the next circle, with a total of three blood spots collected. Care was taken to avoid using too little or too much blood, collecting blood on both sides of the paper simultaneously, or reapplying blood to the same spot. The collection cards with blood samples were placed in a clean, ventilated basket or drawer, away from direct sunlight, ultraviolet light, heat, and volatile chemicals. The spots were allowed to air-dry naturally to a deep brown color, ensuring they were completely dry before stacking to avoid contamination. Once fully dried, each collection card was inspected and sealed individually in a biosafety bag, with care taken not to touch the blood spots during handling. The sealed samples were then stored in a refrigerator at 2–8°C.

### 2.3 Sample processing and analysis

Sample processing and analysis were conducted at Shenzhen Aone Medical Laboratory Co, Ltd. Four 3 mm holes were punched in the blood filter paper and placed in a 96-well filter plate. A total of 100 μl of methanol with amino acid and acylcarnitine isotope internal standards were added and allowed to sit for 20 min. The mixture was then centrifuged into a new polypropylene plate and dried at 50°C. After that, 60 μl of 3 mol/L hydrochloric acid in n-butanol were added, covered with a PTFE membrane, and incubated at 65°C for 15 min. The mixture was dried again at 50°C, and the residues were dissolved in 100 μl of 80% acetonitrile, then covered with aluminum foil. The mobile phase was set to 80% acetonitrile and established for Mass Spectrometry analysis, with flow rates adjusted to 140 μl/min for 0.2 min, 30 μl/min for 1 min, and 300 μl/min for 0.2 min. The autosampler injected 20 μl per sample. Detection was performed using multiple reaction monitoring and neutral loss scanning from 140 to 280 m/z (neutral loss fragment m/z of 102), with each sample analyzed for about 2 min (API 3200 mass spectrometer produced by AB Sciex).

### 2.4 Statistical analysis

Statistical analysis and graphing were performed using SPSS v. 23.0 (SPSS Inc., Chicago, Illinois) and R (version 4.2.3) (https://www.rstudio.com/), with a significance level set at |log2(FC)| > 0.38 (|FC| > 1.3) and *P* < 0.05. For normally distributed data, results are presented as mean ± standard deviation; comparisons between two groups were made using the *t*-test and Chi-square test. For non-normally distributed data, results are presented as median (M) with interquartile range (Q1, Q3); comparisons between two groups were made using the Mann–Whitney *U* test and the chi-square test.

The concentrations of amino acids (AAs) and carnitines (μmol/L) obtained from LC-MS/MS analysis were preprocessed and standardized. Missing data in the dataset were filtered and imputed using the mean value of the respective variables. Metabolite quantities were log10 transformed and visually inspected for normality. Metabolites with mean and median peak intensities below the mass spectrometer's limit of detection were removed from further analysis to ensure robust statistical comparisons between clinical groups. Fold changes between groups were calculated and log2 transformed (log2FC) for visualization. Cluster analysis and heatmap visualization were conducted using the pheatmap package in R (21). Principal component analysis (PCA) was performed using the stats package, and partial least squares discriminant analysis (PLS-DA) was carried out using the mixOmics package (22). In the PLS-DA model, metabolites with a variable importance in projection (VIP) score >1 were deemed significant, with *p* < 0.05 in univariate analysis. Based on VIP score, *p*-value, adjusted *p*-value (bonferroni-hochberg adjusted *p*-value), and log2FC, we selected variables with VIP > 1, adjusted *p*-value < 0.05, and significant log2FC as candidate biomarkers. A volcano plot was generated using the EnhancedVolcano package (23). Metabolic biomarkers with the best sensitivity and specificity for assessing BPD risk were selected through receiver operating characteristic (ROC) analysis. The ROC curve plots sensitivity against 1-specificity for each possible cutoff value, and the AUC quantifies the overall diagnostic accuracy. An AUC value of 0.5 indicates no predictive ability, while values closer to 1 represent better discrimination.

## 3 Results

### 3.1 Basic clinical characteristics of the enrolled preterm infants

**Figure 1:**
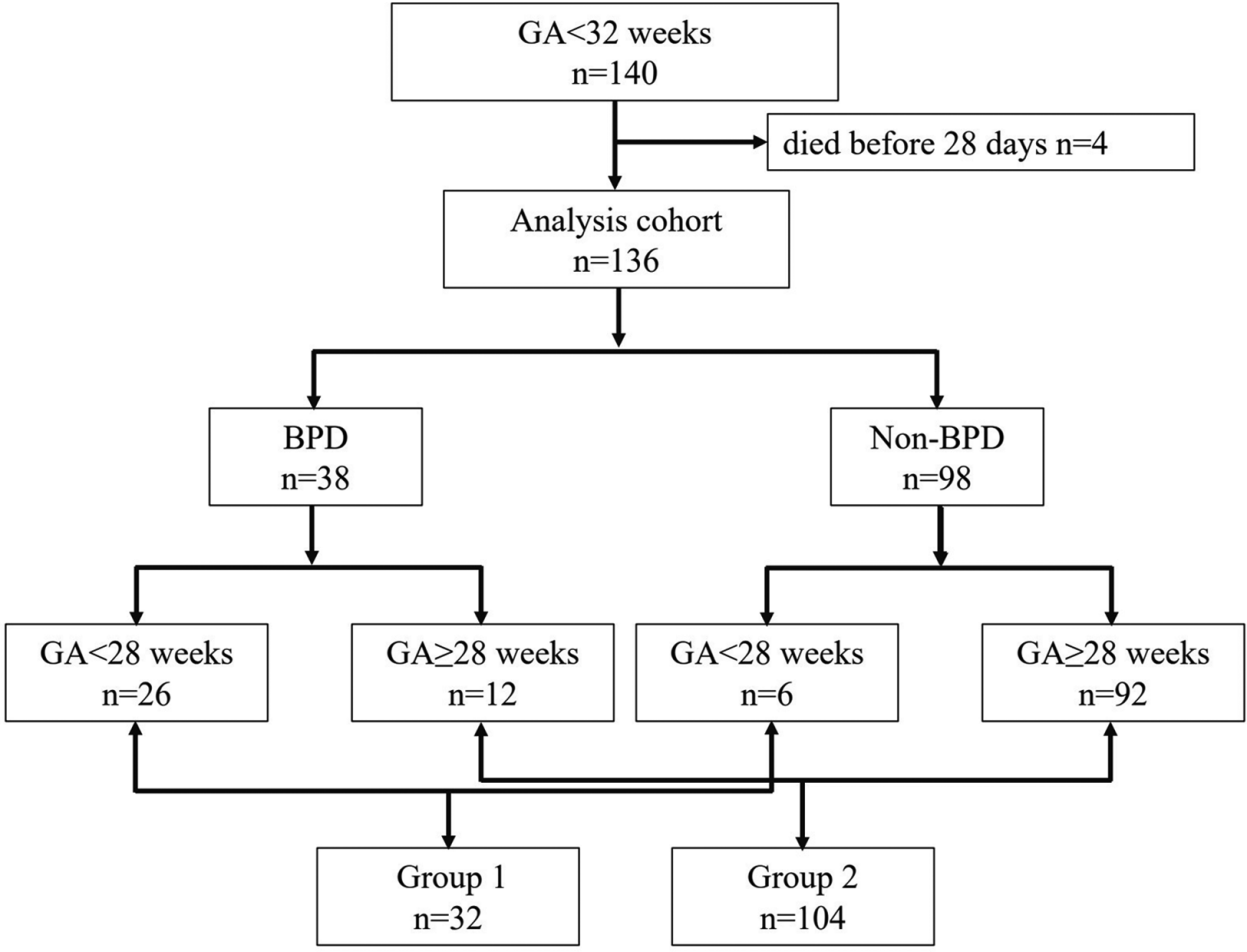
Flow chart for the population under study.

DBS samples from 140 infants were used in the study. Four neonates who died before 28 days were excluded because they did not survive long enough to meet the diagnostic criteria for BPD at 36 weeks postmenstrual age. Among the remaining 136 neonates, 38 developed BPD, and 98 did not (see Figure 1). Infants with BPD had significantly lower gestational ages and birth weights (*P* < 0.05), as well as a higher incidence of small for gestational age (SGA) and longer Length of hospital stay (LOS) (*P* < 0.05), as shown in Table 1. To control for the significant differences in GA and developmental variations, we conducted a subgroup analysis. Since the minimum gestational age in the study population was 24^+4^ weeks, we divided the data into two groups: 24–27^+6^ weeks (Group 1) and 28–31^+6^ weeks (Group 2). From Table 1, it is evident that there were no significant differences in GA, Birth Weight (BW), SGA and LOS between the BPD and non-BPD groups in Group 1. However, in Group 2, infants with BPD showed significantly lower GA and birth weights (*P* < 0.05). Heatmaps, PCA, and PLS-DA were conducted for metabolites within each group. By constructing and validating a predictive classification model, we explored the relationship between BPD and patterns of circulating biomarkers.

**Table 1:** Demographic characteristics of the neonates.

Variable	All neonates recruited	Neonates with BPD	Neonates without BPD	t/Z/*χ*^2^	*P-value*
Neonates, *N* (%)	136	38 (27.94)	98 (72.06)		
Maternal age(years)	31.93 ± 4.63	32.21 ± 4.75	31.83 ± 4.60	−0.43	0.67
IVF baby, *N* (%)	48 (35.29)	12 (31.58)	36 (36.73)	0.32	0.57
ACU, *N* (%)	125 (91.91)	33 (86.84)	92 (93.88)	1.82	0.18
Chorioamnionitis, *N* (%)	53 (38.97)	19 (50.00)	34 (34.69)	2.70	0.10
GH, *N* (%)	29 (21.32)	12 (31.58)	17 (17.35)	3.31	0.07
GDM, *N* (%)	38 (27.94)	11 (28.95)	27 (27.55)	0.03	0.87
GA (weeks)	29.78 (28.11, 30.89)	27.07 (26.14, 28.14)	30.43 (29.29, 31.25)	−7.71	0.00
BW (grams)	1,220.74 ± 380.37	824.21 ± 158.81	1,374.49 ± 325.98	13.16	0.00
SGA, *N* (%)	11 (8.09)	6 (15.79)	5 (5.10)	4.21	0.04
LOS (days)	49.50 (36.75, 70.00)	76.50 (69.25, 92.5)	40.50 (34.00, 50.75)	−8.16	0.00
Variable	Subgroups	Neonates with BPD	Neonates without BPD	t/Z/*χ*^2^	*P-value*
Neonates, *N* (%)	Neonates with GA 24–27^+6^ weeks (32)	26 (81.25)	6 (18.75)		
GA (weeks)	26.50 (25.57, 27.43)	26.43 (25.39, 27.21)	27.57 (26.43, 27.75)	−1.87	0.62
BW (grams)	796.25 ± 142.46	773.46 ± 133.54	895.00 ± 149.23	−1.97	0.58
SGA, *N* (%)	3 (9.38)	2 (7.69)	1 (16.67)	0.45	0.49
LOS (days)	83.59 ± 19.09	86.73 ± 18.79	70.00 ± 14.87	−2.03	0.051
Neonates, *N* (%)	Neonates with GA 28–31^+6^ weeks (104)	12 (11.54)	92 (88.46)		
GA (weeks)	30.42 (29.29,31.14)	28.71 (28.18,29.21)	30.50 (29.61,31.29)	−3.98	0.00
BW (grams)	1,351.35 ± 332.14	934.17 ± 158.20	1,405.76 ± 309.67	−5.17	0.00
SGA, *N* (%)	8 (7.69)	4 (33.33)	4 (43.48)	8.81	0.00
LOS (days)	41.00 (34.75, 52.00)	71.50 (63.00,77.50)	40.00(32.75, 50.00)	−5.10	0.00

Note: BPD, bronchopulmonary dysplasia; N, number; IVF, *in vitro* fertilization; ACU, antenatal corticosteroid use; GH, gestational hypertension; GDM, gestational diabetes mellitus; GA, gestational age; BW, birth weight; SGA, small for gestational age; LOS, length of hospital stay.

### 3.2 Metabolic differences between infants with BPD and non-BPD

**Figure 2:**
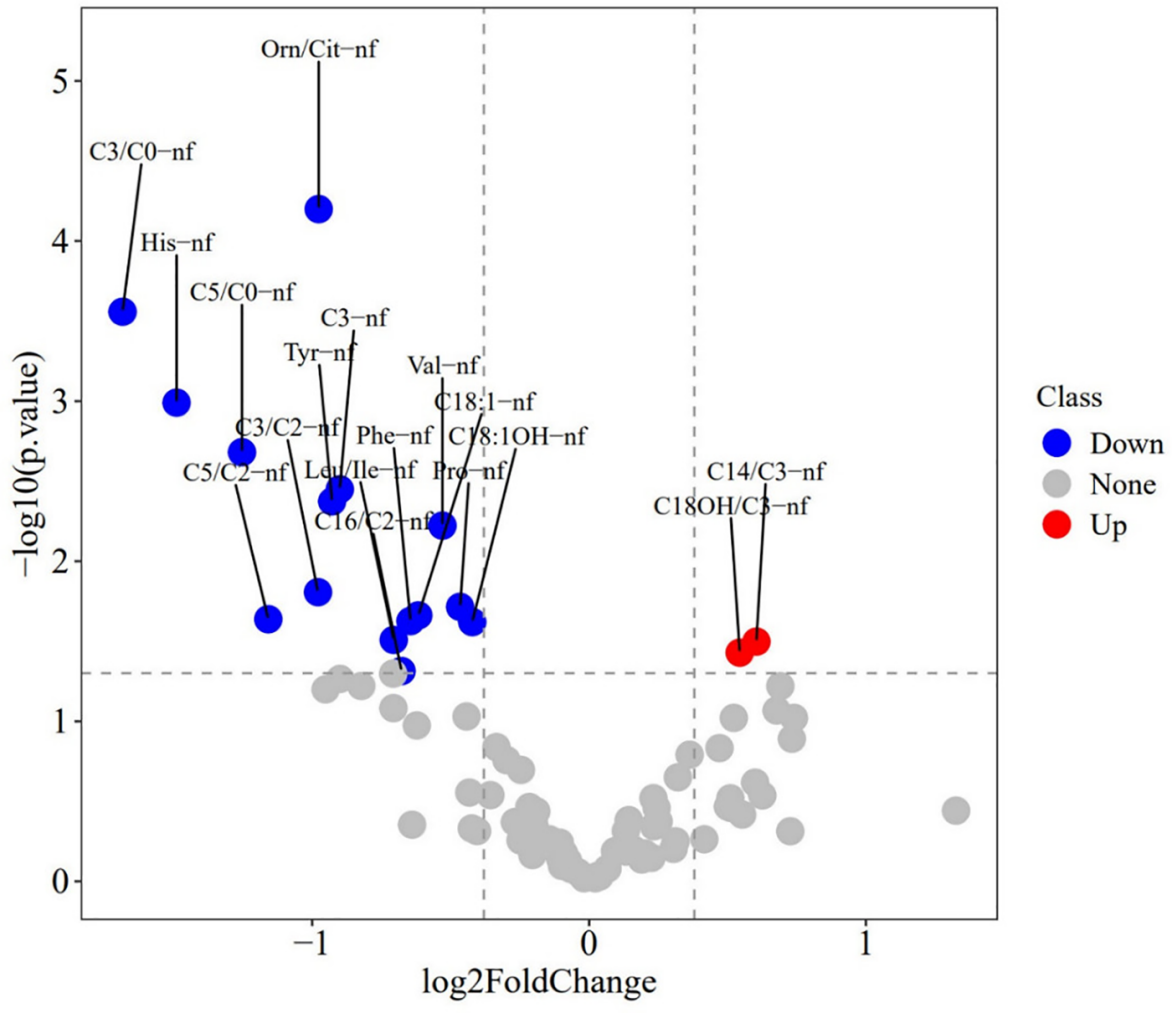
Volcano plot of metabolites in 32 preterm infants (GA of 24–27^+6^ weeks). The size of the solid circles represents the VIP (Variable Importance in Projection) value; the larger the VIP value, the larger the point, indicating a greater contribution of the metabolite to the model. Red points on the right indicate metabolites that are upregulated in the BPD group, while blue points on the left indicate metabolites that are downregulated in the BPD group. Gray points represent metabolites with no significant change.

To identify differential metabolites between the BPD and non-BPD groups, we used R packages to perform sequential analyses including heatmap, PCA, PLS-DA, and volcano plot analysis on the metabolites in each group. The heatmap reveals a consistent distribution of metabolites across both the BPD and non-BPD groups (see Supplementary Figure S1). Based on the component analysis of the PCA plot, no significant discernible differences were found between the BPD group and non-BPD group. (see Supplementary Figure S2). PLS-DA analysis of all metabolites revealed that the differences between BPD and No BPD in Group 1 were significantly larger than those in the other two groups (see Supplementary Figure S3). The BER plot generated from the PLS-DA results indicated that a low BER was achieved using 2 components with the “mahalanobis.dist” algorithm. However, due to the smaller sample size, we opted for the superior “max.dist” algorithm (see Supplementary Figure S4). Simultaneously, based on VIP scores ranking the top 16 metabolites on the *x*-axis, we arranged the most discriminative metabolites in descending order of coefficient scores (see Table 2). Based on the volcano plot of 32 preterm infants with gestational ages between 24 and 27^+6^ weeks, we identified 17 differential metabolites: Tetradecanoylcarnitine(C14)/Propionylcarnitine(C3), Hydroxyoctadecanoylcarnitine(C18-OH)/C3, Histidine, Leucine/Isoleucine, Phenylalanine, Proline, Tyrosine, Valine, Ornithine/Citrulline, C3, Octadecenoylcarnitine (C18:1), Hydroxyoctadecenoylcarnitine (C18:1-OH), C3/Carnitine (C0), C3/Acetylcarnitine (C2), Valerylcarnitine (C5)/C0, C5/C2, and Hexadecanoylcarnitine (C16)/C2 (see Figure 2). Among these, only Ornithine/Citrulline, C3/C0, Histidine, and C5/C0 met the criteria (see Table 2), showing upregulation in the non-BPD group and downregulation in the BPD group (*P* < 0.05).

**Table 2:** Analysis of Key indicators for 16 differential metabolites in 32 preterm infants (GA of 24–27^+6^ weeks).

Metabolite	VIP	*P*-value	Adj. *P*-value	FC	log2FC
Orn/Cit	1.88	0.000	0.005*	0.51	−0.98
Leu/Ile	1.74	0.031	0.184	0.61	−0.71
C5/C0	1.74	0.002	0.045*	0.42	−1.25
Val	1.65	0.006	0.074	0.69	−0.53
C3/C2	1.62	0.016	0.160	0.51	−0.98
Tyr	1.60	0.004	0.061	0.53	−0.93
Phe	1.58	0.024	0.160	0.64	−0.65
C3/C0	1.47	0.000	0.012*	0.31	−1.68
C3	1.37	0.004	0.061	0.54	−0.90
His	1.32	0.001	0.030*	0.36	−1.49
Pro	1.21	0.019	0.160	0.72	−0.47
C18-OH	1.20	0.348	0.674	1.18	0.24
C18:1-OH	1.13	0.024	0.160	0.75	−0.42
C14/C3	1.11	0.032	0.184	1.52	0.60
C18:1	1.08	0.022	0.160	0.65	−0.62
C5/C2	0.96	0.023	0.160	0.45	−1.16

Note: VIP, variable importance in projection; Adj. *P*-Value*, Bonferroni-Hochberg adjusted *P*-value; FC, fold change; Orn, ornithine; Cit, citrulline; Leu, leucine; Ile, isoleucine; C5, valerylcarnitine; C0, carnitine; Val, valine; C3, propionylcarnitine; C2, acetylcarnitine; Tyr, tyrosine; Phe, phenylalanine; His, histidine; Pro, proline; C18-OH, hydroxyoctadecanoylcarnitine; C18:1-OH, hydroxyoctadecenoylcarnitine; C14, tetradecanoylcarnitine; C18:1, octadecenoylcarnitine.

* *p* < 0.05.

### 3.3 The value of metabolic biomarkers in predicting BPD

**Figure 3:**
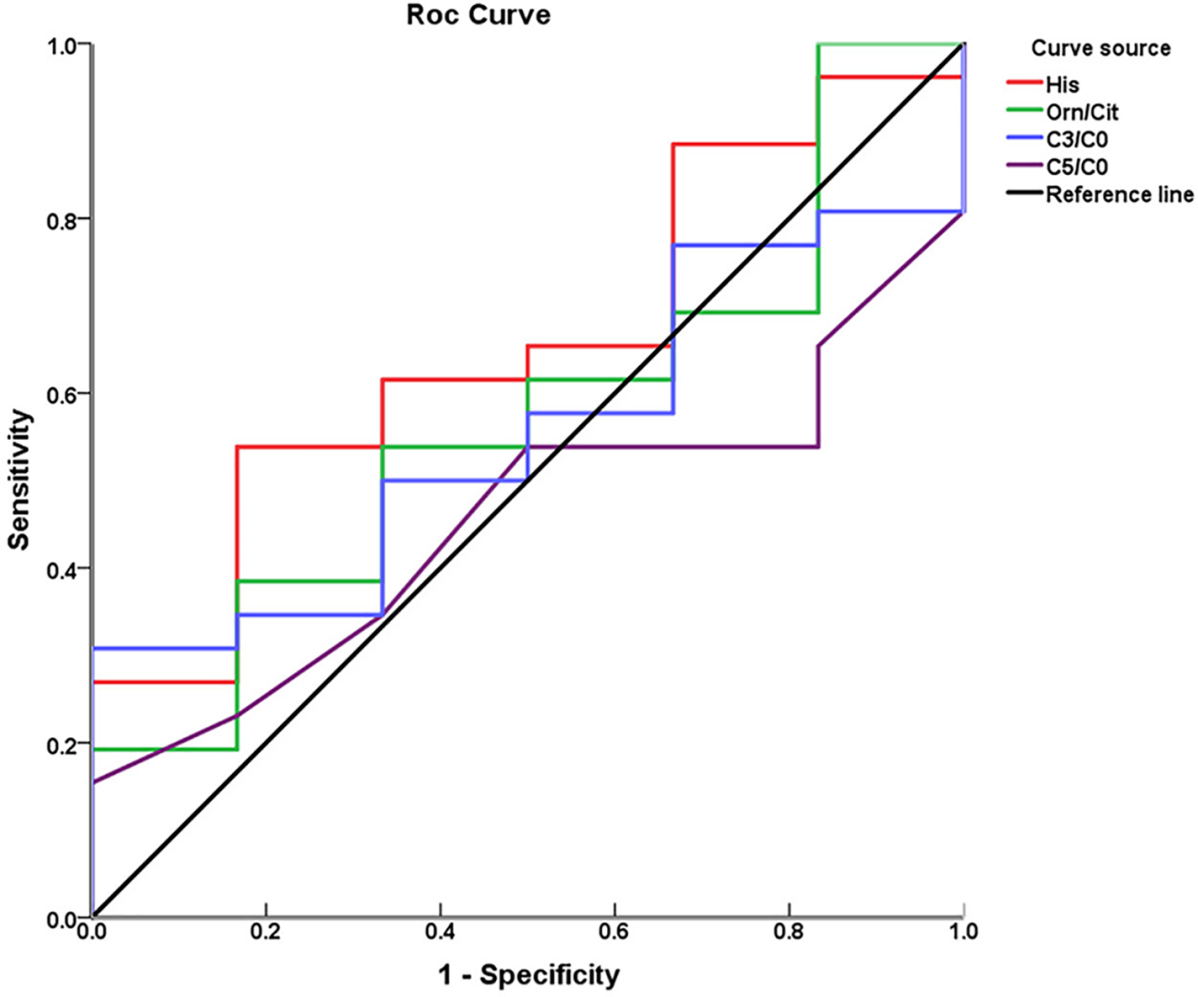
Receiver operating characteristic curves of individual metabolic biomarkers for predicting BPD.

Through ROC curve analysis, the performance of the three metabolic markers in predicting BPD was generally moderate, with all the area under the curve (AUC) values greater than 0.5. Histidine had the highest AUC value at 0.65, indicating a potential predictive ability for the occurrence of BPD, as shown in Figure 3; Table 3.

**Table 3:** Concentrations of four differential metabolites (µmol/L) and their early predictive value for bronchopulmonary dysplasia.

Metabolites	BPD(*N* = 26)	No BPD(*N* = 6)	AUC	*P-value*	95% CI
His	13.74 (11.5,40.4)	19.80 (13.98,76.76)	0.65	0.25	0.43–0.88
Orn/Cit	3.82 (2.41,5.57)	4.12 (2.78,6.72)	0.57	0.60	0.33–0.81
C3/C0	0.048 (0.019,0.064)	0.052 (0.029,0.065)	0.55	0.70	0.34–0.77
C5/C0	0.005 (0.004,0.008)	0.006 (0.004,0.007)	0.46	0.74	0.25–0.67

Note: BPD, bronchopulmonary dysplasia; N, number; AUC, area under the curve; CI, credibility interval; His, histidine; Orn, ornithine; Cit, citrulline; C3, propionylcarnitine; C5, valerylcarnitine; C0, carnitine.

## 4 Discussion

This study is a single-center observational cohort study that enrolled premature infants with GA < 32 weeks. BPD in preterm infants was diagnosed according to the 2018 NICHD criteria (20). The study found the incidence rate of BPD to be 27.94%, which is consistent with results from current large-scale multicenter studies (24). This study found that infants with BPD have significantly lower GA and BW (*P* < 0.05) (see Table 1). To control for the significant differences in GA, subgroup analysis of the data was performed. In Group 1, there were no significant differences in GA, BW, SGA and LOS between the BPD and Non-BPD groups (*P* > 0.05) (see Table 1).

This study builds on our previous research, utilizing the simplicity and ease of storage of DBS. We collected venous blood DBS within 24 h of birth and used LC-MS/MS to analyze 86 metabolites. The results reveal significant metabolic differences in amino acids and carnitines between infants with BPD and those without. Specifically, histidine (log2FC = −1.49, adj. *p*-value = 0.030) and ornithine/citrulline (log2FC = −0.98, adj. *p*-value = 0.005) among the amino acids, and C3/C0 (log2FC = −1.68, adj. *p*-value = 0.012) and C5/C0 (log2FC = −1.25, adj. *p*-value = 0.045) among the acylcarnitines, were significantly lower in infants with BPD.

Metabolic homeostasis influences various cellular bioenergetic processes, including cell proliferation, differentiation, autophagy, and apoptosis, all critical in the pathogenesis of chronic lung diseases such as BPD (13, 25, 26). Research indicates that infants with BPD exhibit metabolic abnormalities, which have been further associated with mitochondrial respiratory dysfunction (27–29). This study identified a significant reduction in the levels of histidine and the ornithine/citrulline ratio in the blood of BPD infants. These findings corroborate previous research. Course et al. (30) similarly observed decreased histidine and ornithine concentrations in exhaled condensates from BPD patients.

Histidine, as an essential amino acid, is a fundamental building block for protein synthesis and plays a vital role in cellular growth and repair. Additionally, histidine exhibits significant anti-inflammatory effects by inhibiting the activation of nucleotide-binding oligomerization domain-like receptor (NLR) family pyrin domain-containing 3 (NLRP3) inflammasomes, thereby mitigating lung inflammation (31). Lung inflammation and oxidative stress are two primary pathological mechanisms in the occurrence and development of BPD. Histidine effectively enhances the antioxidant capacity of the glutathione system by reducing levels of hydrogen peroxide and malondialdehyde, increasing antioxidant enzyme activity, and elevating levels of glutamate and cysteine to promote endogenous glutathione synthesis (32, 33). Currently, there are no studies investigating the impact of histidine supplementation on BPD in premature infants. In our study, we observed lower histidine levels in the blood of BPD premature infants born <28 weeks within 24 h of birth. This may be attributed to increased histidine consumption in extremely low gestational age newborns during the early catabolic stress phase of life.

Ornithine is a non-essential amino acid produced as an intermediate in the urea cycle. It serves as a crucial substrate for the synthesis of polyamines and citrulline, playing an important role in regulating various metabolic processes (34). Polyamines play a crucial role in cell proliferation, differentiation, and tissue repair. Due to incomplete lung development in premature infants, ornithine promotes polyamine synthesis, supporting lung cell proliferation and differentiation, thereby aiding in the repair and development of lung tissue (35, 36). Animal experiment has demonstrated that pulmonary artery endothelial cells increase polyamines under hypoxic conditions (37). In our study, the ratio of ornithine to citrulline in the blood of BPD preterm infants with GA < 28 weeks significantly decreased. This may be associated with the immature lung development in extremely preterm infants and the promotion of ornithine conversion to polyamines under early relative hypoxic conditions. Additionally, ornithine plays a crucial role in the urea cycle, aiding in the elimination of excess ammonia and maintaining cellular homeostasis. In this cycle, ornithine combines with ammonia and carbon dioxide to form citrulline. Citrulline is a non-essential amino acid that can alleviate inflammation and hyperoxic lung injury in preclinical models of BPD. Research has confirmed its protective effect on early lung development in neonatal rats (38). In our cohort of extremely preterm infants with BPD, we observed a decrease in the ratio of ornithine to citrulline, likely due to a more substantial decline in ornithine levels and a less pronounced decrease in citrulline levels.

Another essential nutrient, fatty acids, were dysregulated in neonatal rat models and in rat lung epithelial cells under hyperoxic conditions (39). Carnitines, comprising free carnitines and acylcarnitines, serve as essential biomarkers in fatty acid metabolism. Free carnitines are crucial in the conversion of fatty acids into acylcarnitines and their subsequent transport into mitochondria for further energy production via β-oxidation. Evaluating carnitine patterns in infants with BPD may reveal alterations in fatty acid metabolism linked to the disease's pathogenesis. In animal models, L-carnitine supplementation has been shown to reduce hyperoxia-induced apoptosis and lung injury (38). Peterson et al. (40) discovered that neonatal hyperoxia decreased carnitine and acylcarnitine levels in mouse lungs. In our study, we observed a significant reduction in acylcarnitine C3/C0 and C5/C0 levels among extremely preterm infants with BPD. In our ROC analysis, we examined the predictive performance of four key metabolites in relation to BPD. Among the metabolites, the AUC value for histidine suggests that it may have some predictive ability for BPD occurrence, but it is not a strong standalone predictor. Further studies in larger cohorts are needed to assess its diagnostic relevance.

Due to the complex pathophysiology of BPD, it is insufficient to rely on a single marker for comprehensive assessment and prediction of its development. Developing and utilizing indicators linked to specific clinical conditions is critical for early identification of high-risk newborns predisposed to BPD. The metabolome, encompassing all metabolites, reflects the interaction of pathophysiological states and environmental stimuli, thus enabling the identification of metabolites associated with specific etiologies. Currently, metabolic patterns of BPD have been studied in various biological fluids, such as amniotic fluid, umbilical cord blood, peripheral blood, tracheal aspirate, and urine. Baraldi et al. (41) conducted a metabolic analysis of amniotic fluid samples and found that preterm infants with higher levels of leucine, hydroxy fatty acids, and oxo-fatty acids, and lower levels of S-adenosylmethionine, were more likely to develop BPD. This may be linked to oxidative stress. La Frano et al. (42) conducted a metabolomics study on umbilical cord blood from preterm infants born at less than 32 weeks gestation. They observed a negative correlation between phosphatidylcholine levels in umbilical cord blood and the severity of BPD, suggesting potential immaturity in lipid biosynthesis as a contributing factor. Piersigilli et al. (43) analyzed antioxidant metabolite levels in tracheal aspirates from premature infants with BPD one week after birth. Their findings revealed elevated levels of histidine, glutamate, citrulline, glycine, isoleucine, as well as acylcarnitine C16-OH and C18:1-OH compared to non-BPD infants. This is inconsistent with our study results, possibly because the oxidative stress process in newborns has not fully manifested, particularly in the lungs. Additionally, factors such as oxygen therapy, inflammation, or oxidative stress may activate metabolic pathways and compensatory mechanisms at an early stage, which could affect histidine levels differently in the blood and lung tissue. Furthermore, Fanos et al. (44) collected urine samples from infants born at 29 weeks of gestation, while Pintus et al. (45) analyzed urine samples from infants born at less than 28 weeks of gestation on their seventh day of life for metabolite analysis. Both studies found that the levels of lactate and trimethylamine N-oxide (TMAO) in the urine of BPD infants showed opposite trends. Currently, there is no consensus on the metabolic markers for BPD. This may be due to differences in the types of biological fluids analyzed, the timing of sample collection, and specific clinical conditions such as gestational age, sex, and type of nutrition (enteral or parenteral). In our study, we analyzed amino acid and fatty acid patterns within the first 24 h after birth, specifically excluding the influence of enteral and parenteral nutrition. Although biomarkers in tracheal aspirates can more accurately reflect lung conditions in the development of BPD, these markers are not routinely obtained, particularly in healthy newborns without respiratory support. Therefore, we collected DBS from the enrolled infants within the first 24 h after birth, as they are easily obtainable and minimally invasive.

This study has its limitations. We conducted targeted metabolomics analysis using samples collected within the first 24 h after birth. The limited variety of detected metabolites prevented the identification of significant metabolic pathways, potentially overlooking important metabolites. Furthermore, our subgroup analyses were restricted to gestational age and birth weight due to the small sample size, without precise adjustment for other influencing factors. Therefore, based on the preliminary results of this study, our research team has collaborated with the Shenzhen Neonatal Data Network to establish a prospective, multicenter, large-scale, long-term follow-up cohort of infants born preterm at less than 32 weeks of gestation. This cohort includes repositories of biological samples such as blood, urine, stool, and breast milk collected at multiple time points post-birth. Our aim is to analyze the metabolic profiles of preterm infants under different nutritional conditions, identify and validate early predictive biomarkers for BPD, and integrate various biological samples and clinical indicators to develop a metabolomics-based predictive model for the onset and progression of BPD. Ultimately, our goal is for this approach to eventually be applied in clinical practice, enabling early identification of high-risk infants. This could lead to closer monitoring and tailored respiratory management strategies, potentially reducing exposure to known BPD risk factors (e.g., prolonged mechanical ventilation and oxygen therapy). As a result, this may lower the incidence of severe BPD in preterm infants in our country and improve both short- and long-term outcomes.

## 5 Conclusions

Through metabolic profiling of DBS collected within 24 h after birth from preterm infants with BPD, we identified four differential metabolites in those with—GA < 28 weeks: histidine, ornithine/citrulline, C3/C0, and C5/C0. These findings suggest that histidine may have potential value in the early prediction of BPD in preterm infants with GA < 28 weeks.

## Data Availability

The original contributions presented in the study are included in the article/Supplementary Material, further inquiries can be directed to the corresponding author.

## References

[1] Zhu Z Yuan L Wang J Li Q Yang C Gao X Mortality and morbidity of infants born extremely preterm at tertiary medical centers in China from 2010 to 2019. JAMA Netw Open. (2021) 4:e219382. 10.1001/jamanetworkopen.2021.9382 33974055 PMC8114138

[2] Cao Y Jiang S Sun J Hei M Wang L Zhang H Assessment of neonatal intensive care unit practices, morbidity, and mortality among very preterm infants in China. JAMA Netw Open. (2021) 4:e2118904. 10.1001/jamanetworkopen.2021.18904 34338792 PMC8329742

[3] Bell EF Hintz SR Hansen NI Bann CM Wyckoff MH DeMauro SB Mortality, in-hospital morbidity, care practices, and 2-year outcomes for extremely preterm infants in the US, 2013–2018. JAMA. (2022) 327:248–63. 10.1001/jama.2021.23580 35040888 PMC8767441

[4] Jeon GW Oh M Chang YS. Definitions of bronchopulmonary dysplasia and long-term outcomes of extremely preterm infants in Korean neonatal network. Sci Rep. (2021) 11:24349. 10.1038/s41598-021-03644-7 34934085 PMC8692520

[5] Jeon GW Oh M Lee J Jun YH Chang YS. Comparison of definitions of bronchopulmonary dysplasia to reflect the long-term outcomes of extremely preterm infants. Sci Rep. (2022) 12:18095. 10.1038/s41598-022-22920-8 36302832 PMC9613988

[6] Katz TA Vliegenthart R Aarnoudse-Moens C Leemhuis AG Beuger S Blok GJ Severity of bronchopulmonary dysplasia and neurodevelopmental outcome at 2 and 5 years corrected age. J Pediatr. (2022) 243:40–46.e2. 10.1016/j.jpeds.2021.12.018 34929243

[7] Katz TA van Kaam AH Schuit E Mugie SM Aarnoudse-Moens C Weber EH Comparison of new bronchopulmonary dysplasia definitions on long-term outcomes in preterm infants. J Pediatr. (2023) 253:86–93.e4. 10.1016/j.jpeds.2022.09.022 36150504

[8] Jensen EA Edwards EM Greenberg LT Soll RF Ehret D Horbar JD. Severity of bronchopulmonary dysplasia among very preterm infants in the United States. Pediatrics. (2021) 148(1):e2020030007. 10.1542/peds.2020-030007 34078747 PMC8290972

[9] Gilfillan M Bhandari A Bhandari V. Diagnosis and management of bronchopulmonary dysplasia. BMJ. (2021) 375:n1974. 10.1136/bmj.n1974 34670756

[10] Hwang JS Rehan VK. Recent advances in bronchopulmonary dysplasia: pathophysiology, prevention, and treatment. Lung. (2018) 196:129–38. 10.1007/s00408-018-0084-z 29374791 PMC5856637

[11] Shukla VV Ambalavanan N. Recent advances in bronchopulmonary dysplasia. Indian J Pediatr. (2021) 88:690–5. 10.1007/s12098-021-03766-w 34018135

[12] Chen YT Lan HY Tsai YL Wu HP Liaw JJ Chang YC. Effects of bradycardia, hypoxemia and early intubation on bronchopulmonary dysplasia in very preterm infants: an observational study. Heart Lung. (2024) 65:109–15. 10.1016/j.hrtlng.2024.02.009 38471331

[13] Sun T Yu H Li D Zhang H Fu J. Emerging role of metabolic reprogramming in hyperoxia-associated neonatal diseases. Redox Biol. (2023) 66:102865. 10.1016/j.redox.2023.102865 37659187 PMC10480540

[14] Bonadies L Moschino L Valerio E Giordano G Manzoni P Baraldi E. Early biomarkers of bronchopulmonary dysplasia: a quick Look to the state of the art. Am J Perinat. (2022) 39:S26–30. 10.1055/s-0042-1758867 36470296

[15] Hocq C Vanhoutte L Guilloteau A Massolo AC Van Grambezen B Carkeek K Early diagnosis and targeted approaches to pulmonary vascular disease in bronchopulmonary dysplasia. Pediatr Res. (2022) 91:804–15. 10.1038/s41390-021-01413-w 33674739

[16] Piersigilli F Bhandari V. Metabolomics of bronchopulmonary dysplasia. Clin Chim Acta. (2020) 500:109–14. 10.1016/j.cca.2019.09.025 31689411

[17] Zhang A Sun H Wang P Han Y Wang X. Modern analytical techniques in metabolomics analysis. Analyst. (2012) 137:293–300. 10.1039/C1AN15605E 22102985

[18] Noto A Fanos V Dessì A. Advances in clinical chemistry. Adv Clin Chem. (2016) 74:35–61. 10.1016/bs.acc.2015.12.006 27117660

[19] Fanos V Pintus R Dessì A. Clinical metabolomics in neonatology: from metabolites to diseases. Neonatology. (2018) 113:406–13. 10.1159/000487620 29852484

[20] Higgins RD Jobe AH Koso-Thomas M Bancalari E Viscardi RM Hartert TV Bronchopulmonary dysplasia: executive summary of a workshop. J Pediatr. (2018) 197:300–8. 10.1016/j.jpeds.2018.01.043 29551318 PMC5970962

[21] Kolde R. Pheatmap: Pretty Heatmaps. San Francisco, CA: GitHub (2018). R package version 1.0.12. Available online at: https://github.com/raivokolde/pheatmap

[22] Rohart F Gautier B Singh A Lê CK. Mixomics: an R package for ‘omics feature selection and multiple data integration. PLoS Comput Biol. (2017) 13:e1005752. 10.1371/journal.pcbi.1005752 29099853 PMC5687754

[23] Blighe K Rana S Lewis M. EnhancedVolcano: Publication-ready volcano Plots with Enhanced Colouring and Labeling. San Francisco, CA: GitHub (2024). R package version 1.22.0. Available online at: https://github.com/kevinblighe/EnhancedVolcano

[24] Li T Zhang G Li R He S Zhang F Yan X Survival and morbidity in very preterm infants in Shenzhen: a multi-center study. Front Pediatr. (2024) 11:1298173. 10.3389/fped.2023.1298173 38464983 PMC10920349

[25] Yan P Liu J Li Z Wang J Zhu Z Wang L Glycolysis reprogramming in idiopathic pulmonary fibrosis: unveiling the mystery of lactate in the lung. Int J Mol Sci. (2023) 25(1):315. 10.3390/ijms25010315 38203486 PMC10779333

[26] Ho TT Warr MR Adelman ER Lansinger OM Flach J Verovskaya EV Autophagy maintains the metabolism and function of young and old stem cells. Nature. (2017) 543:205–10. 10.1038/nature21388 28241143 PMC5344718

[27] Yue L Lu X Dennery PA Yao H. Metabolic dysregulation in bronchopulmonary dysplasia: implications for identification of biomarkers and therapeutic approaches. Redox Biol. (2021) 48:102104. 10.1016/j.redox.2021.102104 34417157 PMC8710987

[28] Ye C Wu J Reiss JD Sinclair TJ Stevenson DK Shaw GM Progressive metabolic abnormalities associated with the development of neonatal bronchopulmonary dysplasia. Nutrients. (2022) 14(17):3547. 10.3390/nu14173547 36079804 PMC9459725

[29] Jeong J Lee Y Han J Kang E Kim D Kim KS Mitochondrial DNA mutations in extremely preterm infants with bronchopulmonary dysplasia. Gene. (2024) 910:148337. 10.1016/j.gene.2024.148337 38432533

[30] Course CW Lewis PA Kotecha SJ Cousins M Hart K Heesom KJ Evidence of abnormality in glutathione metabolism in the airways of preterm born children with a history of bronchopulmonary dysplasia. Sci Rep. (2023) 13:19465. 10.1038/s41598-023-46499-w 37945650 PMC10636015

[31] Tian Q Xu M He B. Histidine ameliorates elastase- and lipopolysaccharide-induced lung inflammation by inhibiting the activation of the NLRP3 inflammasome. Acta Bioch Bioph Sin. (2021) 53:1055–64. 10.1093/abbs/gmab072 34125142

[32] Thalacker-Mercer AE Gheller ME. Benefits and adverse effects of histidine supplementation. J Nutr. (2020) 150:2588S–92. 10.1093/jn/nxaa229 33000165

[33] Yang P Deng F Yuan M Chen M Zeng L Ouyang Y Metabolomics reveals the defense mechanism of histidine supplementation on high-salt exposure-induced hepatic oxidative stress. Life Sci. (2023) 314:121355. 10.1016/j.lfs.2022.121355 36596407

[34] Sivashanmugam M Jaidev J Umashankar V Sulochana KN. Ornithine and its role in metabolic diseases: an appraisal. Biomed Pharmacother. (2017) 86:185–94. 10.1016/j.biopha.2016.12.024 27978498

[35] Xuan M Gu X Li J Huang D Xue C He Y. Polyamines: their significance for maintaining health and contributing to diseases. Cell Commun Signal. (2023) 21:348. 10.1186/s12964-023-01373-0 38049863 PMC10694995

[36] Thet LA Parra SC. Role of ornithine decarboxylase and polyamines in early postnatal lung growth. J Appl Physiol. (1986) 61:1661–6. 10.1152/jappl.1986.61.5.1661 3096943

[37] Babal P Ruchko M Ault-Ziel K Cronenberg L Olson JW Gillespie MN. Regulation of ornithine decarboxylase and polyamine import by hypoxia in pulmonary artery endothelial cells. Am J Physiol Lung Cell Mol Physiol. (2002) 282:L840–6. 10.1152/ajplung.00347.2001 11880311

[38] Ivanovski N Wang H Tran H Ivanovska J Pan J Miraglia E L-citrulline attenuates lipopolysaccharide-induced inflammatory lung injury in neonatal rats. Pediatr Res. (2023) 94:1684–95. 10.1038/s41390-023-02684-1 37349511

[39] Yao H Gong J Peterson AL Lu X Zhang P Dennery PA. Fatty acid oxidation protects against hyperoxia-induced endothelial cell apoptosis and lung injury in neonatal mice. Am J Resp Cell Mol. (2019) 60:667–77. 10.1165/rcmb.2018-0335OC PMC6543740 30571144

[40] Peterson AL Carr JF Ji X Dennery PA Yao H. Hyperoxic exposure caused lung lipid compositional changes in neonatal mice. Metabolites. (2020) 10(9):340. 10.3390/metabo10090340 32825609 PMC7569933

[41] Baraldi E Giordano G Stocchero M Moschino L Zaramella P Tran MR Untargeted metabolomic analysis of amniotic fluid in the prediction of preterm delivery and bronchopulmonary dysplasia. PLoS One. (2016) 11:e0164211. 10.1371/journal.pone.0164211 27755564 PMC5068788

[42] La Frano MR Fahrmann JF Grapov D Pedersen TL Newman JW Fiehn O Umbilical cord blood metabolomics reveal distinct signatures of dyslipidemia prior to bronchopulmonary dysplasia and pulmonary hypertension. Am J Physiol Lung Cell Mol Physiol. (2018) 315:L870–81. 10.1152/ajplung.00283.2017 30113229 PMC6295510

[43] Piersigilli F Lam TT Vernocchi P Quagliariello A Putignani L Aghai ZH Identification of new biomarkers of bronchopulmonary dysplasia using metabolomics. Metabolomics. (2019) 15:20. 10.1007/s11306-019-1482-9 30830433

[44] Fanos V Pintus MC Lussu M Atzori L Noto A Stronati M Urinary metabolomics of bronchopulmonary dysplasia (BPD): preliminary data at birth suggest it is a congenital disease. J Matern Fetal Neonatal Med. (2014) 27(Suppl 2):39–45. 10.3109/14767058.2014.955966 25284176

[45] Pintus MC Lussu M Dessì A Pintus R Noto A Masile V Urinary ^1^H-NMR metabolomics in the first week of life can anticipate BPD diagnosis. Oxid Med Cell Longev. (2018) 2018:7620671. 10.1155/2018/7620671 30050661 PMC6046120

